# Strategies for minimizing muscle loss during use of incretin‐mimetic drugs for treatment of obesity

**DOI:** 10.1111/obr.13841

**Published:** 2024-09-19

**Authors:** Jeffrey I. Mechanick, W. Scott Butsch, Sandra M. Christensen, Osama Hamdy, Zhaoping Li, Carla M. Prado, Steven B. Heymsfield

**Affiliations:** ^1^ Marie‐Josée and Henry R. Kravis Center for Clinical Cardiovascular Health at Mount Sinai Fuster Heart Hospital and the Division of Endocrinology, Diabetes, and Bone Disease Icahn School of Medicine at Mount Sinai New York New York USA; ^2^ Bariatric and Metabolic Institute Cleveland Clinic Cleveland Ohio USA; ^3^ Integrative Medical Weight Management Seattle Washington USA; ^4^ Harvard Medical School and Joslin Diabetes Center Boston Massachusetts USA; ^5^ Center for Human Nutrition David Geffen School of Medicine, University of California, Los Angeles Los Angeles California USA; ^6^ Department of Agricultural, Food and Nutritional Science University of Alberta Edmonton Canada; ^7^ Pennington Biomedical Research Center of the Louisiana State University System Baton Rouge Louisiana USA

**Keywords:** GLP‐1 receptor agonist, muscle loss, nutrition, resistance training

## Abstract

The rapid and widespread clinical adoption of highly effective incretin‐mimetic drugs (IMDs), particularly semaglutide and tirzepatide, for the treatment of obesity has outpaced the updating of clinical practice guidelines. Consequently, many patients may be at risk for adverse effects and uncertain long‐term outcomes related to the use of these drugs. Of emerging concern is the loss of skeletal muscle mass and function that can accompany rapid substantial weight reduction; such losses can lead to reduced functional and metabolic health, weight cycling, compromised quality of life, and other adverse outcomes. Available evidence suggests that clinical trial participants receiving IMDs for the treatment of obesity lost 10% or more of their muscle mass during the 68‐ to 72‐week interventions, approximately equivalent to 20 years of age‐related muscle loss. The ability to maintain muscle mass during caloric restriction‐induced weight reduction is influenced by two key factors: nutrition and physical exercise. Nutrition therapy should ensure adequate intake and absorption of high‐quality protein and micronutrients, which may require the use of oral nutritional supplements. Additionally, concurrent physical activity, especially resistance training, has been shown to effectively minimize loss of muscle mass and function during weight reduction therapy. All patients receiving IMDs for obesity should participate in comprehensive treatment programs emphasizing adequate protein and micronutrient intakes, as well as resistance training, to preserve muscle mass and function, maximize the benefit of IMD therapy, and minimize potential risks.

## 1 INTRODUCTION

There has been a dramatic increase in the use of the GLP‐1 (glucagon‐like peptide‐1) receptor agonists semaglutide and liraglutide, and the GLP‐1/glucose‐dependent insulinotropic polypeptide (GIP) receptor co‐agonist tirzepatide to treat obesity after clinical trials showed that semaglutide and tirzepatide were highly effective for weight reduction and after FDA approval for obesity.^1^, ^2^ The rapid clinical adoption of these and other incretin‐mimetic drugs (IMDs) for obesity treatment has outpaced the ability of professional medical societies to update clinical practice guidelines. Consequently, important considerations for the use of IMDs, including management of side effects, may receive inadequate attention in the clinic, leaving patients exposed to potential adverse effects and uncertain long‐term outcomes. An emerging concern is loss of skeletal muscle mass and function, an effect that can lead to reduced functional and metabolic health, weight regain or weight cycling, compromised quality of life, and other adverse outcomes.^3^, ^4^ Available evidence provides limited information about preventing or reversing muscle loss associated with the use of IMDs. The goal of this paper is to examine the issue of muscle loss and outline clinical strategies for minimizing the loss of muscle mass and function in people using IMDs for the treatment of obesity. The paper is based on a 1‐day scientific roundtable meeting in August 2023 attended by each of the authors.

## 2 IS THE CONCERN JUSTIFIED?

### 2.1 Muscle loss in people with obesity

Glucagon‐like peptide‐1 and GIP are hormones that exert an incretin effect, in which an oral glucose load induces a stronger insulin response than an isoglycemic intravenous glucose load.^5^ Similar to endogenous incretin hormones, IMDs have pleiotropic effects on the gastrointestinal tract, the central nervous system, and other organ systems. In general, IMDs have a significant effect on the regulation of body weight by suppressing appetite and slowing gastric emptying.^5^ It is widely recognized that restricted caloric intake leading to weight reduction is usually accompanied by loss of muscle mass^6^ and that such loss can be detrimental.^3^, ^4^ Thus, steps to monitor and minimize loss of muscle mass and function are essential components of a comprehensive obesity treatment program.

**FIGURE 1:**
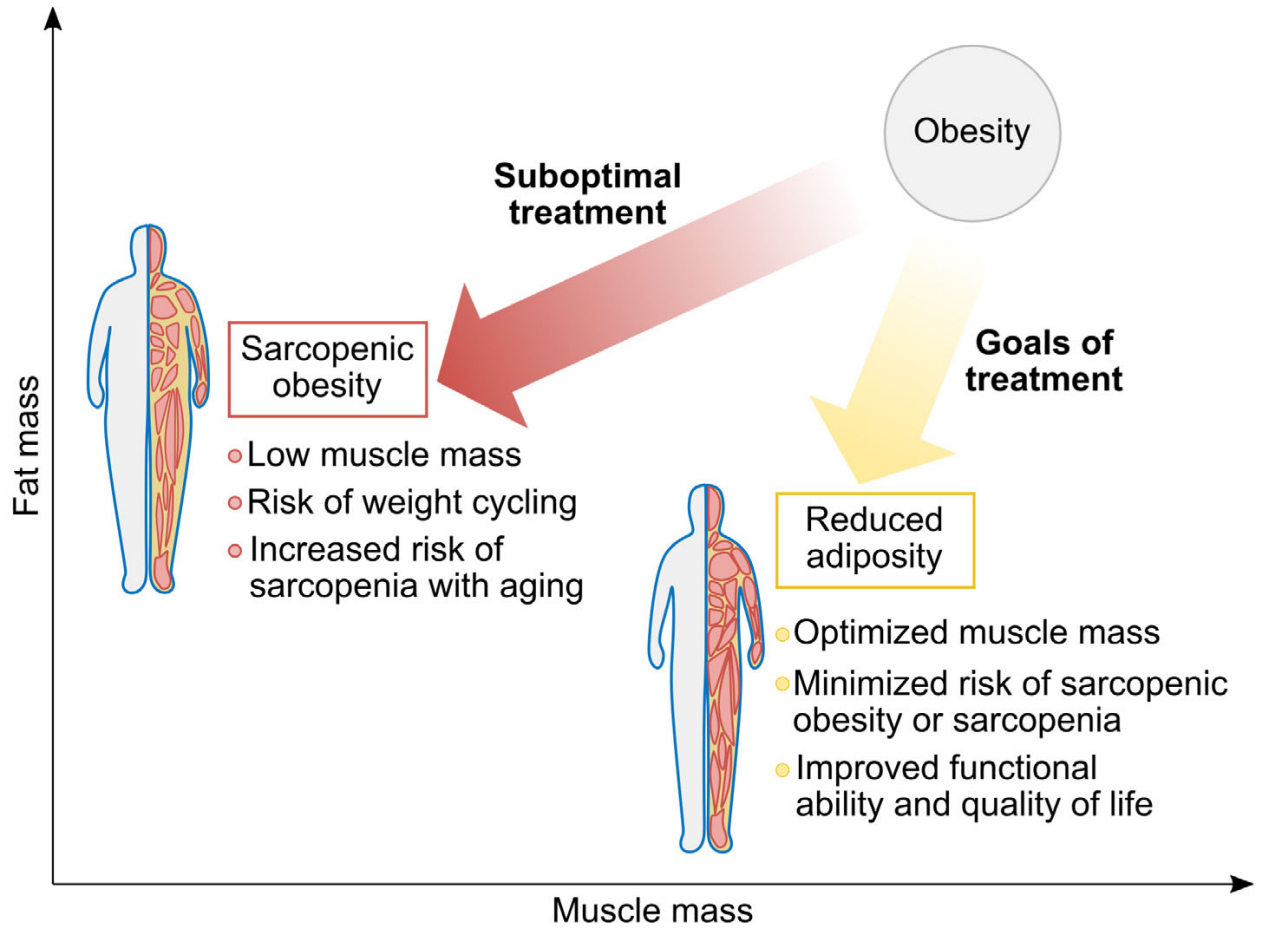
Muscle‐related goals of obesity treatment and muscle‐related complications of suboptimal treatment. Adapted from Prado et al.^7^

**FIGURE 2:**
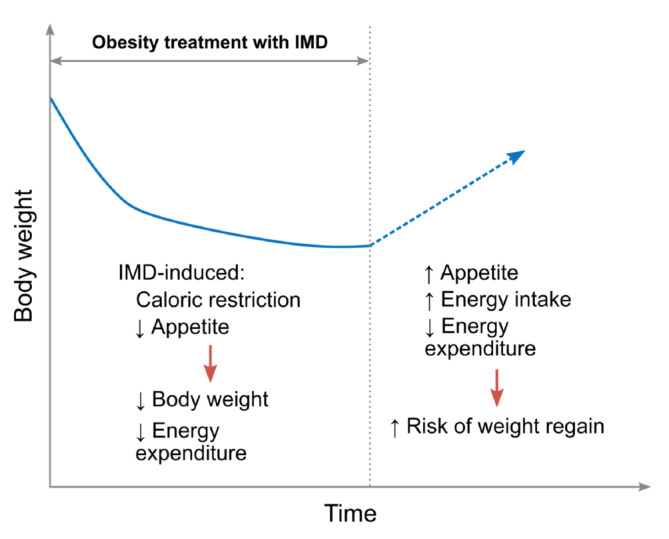
Expected metabolic adaptations during IMD treatment and their relationship with factors associated with increased risk of weight regain after cessation of therapy. Decreased appetite and caloric restriction result in decreased body weight and energy expenditure. Upon cessation of IMD therapy, appetite and energy expenditure often increase, as typically observed after caloric restriction. These changes, along with residual lower energy expenditure due to adaptations such as lower muscle mass, improved muscle efficiency, and reduced basal metabolic rate, may contribute to an increase in the risk of weight regain though more research is needed to fully understand the causes of weight regain.^12^, ^13^

The central goals of obesity treatment are long‐term reduction of excess and abnormal adiposity, as well as complications associated with excess body weight, such as cardiovascular disease (Figure 1).^8^, ^9^ Unfortunately, most patients who use anti‐obesity medications, including IMDs, for this purpose regain much of their former weight after stopping therapy.^10^, ^11^ Specifically, at the end of the weight reduction phase, most patients will have lower energy expenditure owing to various adaptations, including lower muscle mass, reduced basal metabolic rate, and possibly improved muscle efficiency (Figure 2).^13^ Because muscle is more metabolically active than adipose tissue, reductions in muscle mass lead to reduced energy expenditure and consequently reduced energy deficit, rendering weight maintenance more difficult. Furthermore, it is likely the body weight regained after stopping treatment is disproportionately composed of adipose tissue, with little regain of lean mass and little increase in energy expenditure.^14^, ^15^ This process can lead to weight cycling, which is particularly common in young and middle‐aged women (<60 years), who are also the most common type of patients seeking obesity treatment.^16^ Severe weight cycling is associated with an increased risk of muscle loss and sarcopenic obesity,^15^, ^17^ which has been reported in as many as 20% of young women (≥18 years of age) seeking obesity treatment.^18^

On average, people with obesity have a higher absolute skeletal muscle mass and strength than those with a normal weight, especially in weight‐bearing muscles.^9^, ^19^, ^20^ However, a substantial percentage of people with obesity have low muscle mass.^21^ Furthermore, when muscle strength is normalized to body weight, it is lower in people living with obesity.^19^ Another factor is that prediabetes and type 2 diabetes (T2D) are common in people with obesity, and T2D is associated with lower muscle mass and more rapid age‐related declines in muscle mass.^22^, ^23^, ^24^, ^25^ In addition, it is widely recognized that excess and abnormal adiposity has detrimental effects on muscle strength and structure, as well as cardiovascular disease, due to multiple mechanisms, including myosteatosis.^19^, ^26^, ^27^

**FIGURE 3:**
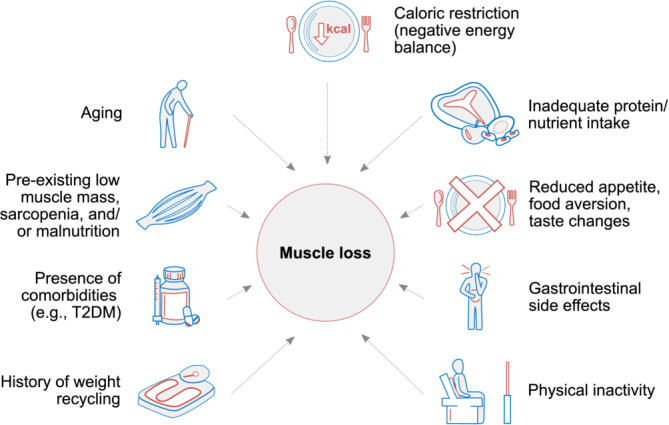
Factors associated with muscle loss that may contribute to, or compound, muscle loss during IMD therapy for obesity. T2DM, type 2 diabetes mellitus.

Low muscle mass is often unrecognized and is independently associated with increased risk of mortality and morbidity, reduced quality of life, increased risk of T2D, and other adverse health outcomes.^3^, ^4^, ^8^, ^28^, ^29^ A recent study estimated that 15.9% of the US adult population (≥20 years old) had obesity with low muscle mass, representing almost 30 million people.^21^ These individuals—and especially women and those with T2D, prediabetes, or metabolic dysfunction‐associated steatotic liver disease (MASLD)—are already at high risk of sarcopenia (age‐related loss of skeletal muscle function and mass),^30^, ^31^ sarcopenic obesity (the co‐existence of excess adiposity and low muscle mass/function, which can occur at any age^32^), and the serious negative health outcomes accompanying those conditions (Figure 3).^16^, ^21^, ^28^, ^29^, ^33^

### 2.2 Muscle loss in phase 3 trials of IMDs for treatment of obesity

**FIGURE 4:**
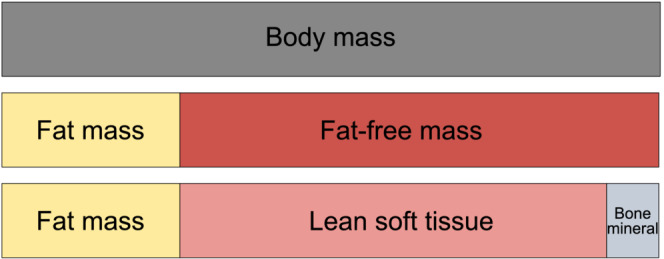
Definitions of body composition terms. Notably, fat‐free mass and lean mass are not synonymous. In many body composition studies, it is unclear whether the values reported as lean mass are consistent with these definitions.

Interpretation of muscle loss in phase 3 trials of IMDs necessitates a clear understanding of body composition terminology and its application. According to precise definitions, lean mass includes lean soft tissues, not bone mass (Figure 4).^34^ Fat‐free mass, in contrast, includes the sum of lean mass and bone mass. Unfortunately, the terms “lean mass” and “fat‐free mass” are often mistakenly used interchangeably, which ignores these critical distinctions. Compounding this issue, many studies fail to explain dual‐energy X‐ray absorptiometry (DXA) methods in sufficient detail, making it challenging to determine which body composition compartment is being assessed and whether the terminology is used accurately.

In a pivotal trial of semaglutide for the treatment of obesity (Semaglutide Treatment Effect in People with Obesity‐1; STEP‐1), a subset of participants had body composition analyzed by DXA before and at the end of the 68‐week treatment period (see supplementary appendix in Wilding et al.^35^). In the semaglutide‐treated (2.4 mg once weekly) group, body weight was reduced by an average of 17.32 kg during the treatment period (vs. 2.65 kg in the placebo group); this included a 6.92‐kg mean reduction in total lean mass (vs. 1.48 kg in the placebo group). Thus, in the semaglutide group, 40% of the reduction in body weight was due to a reduction in lean mass. Perhaps more importantly, 6.92 kg represents 13.2% of the average total lean mass at baseline (52.4 kg). Although muscle usually accounts for about one‐half of lean mass, it is not possible to determine from this publication the actual proportion of lean mass that was muscle versus other non‐fat tissues such as the liver, heart, and other organs and tissues. However, it is reasonable to conclude that muscle loss accounted for at least half of the reduction in lean mass during the STEP‐1 trial.^36^, ^37^, ^38^ On this basis, a conservative estimate is that participants in the semaglutide group lost at least 10% of their muscle mass during the 68‐week treatment period.

**FIGURE 5:**
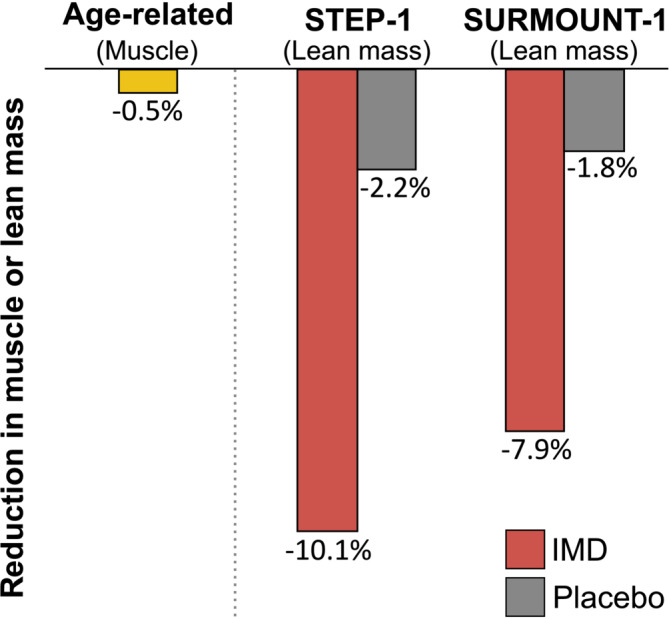
Estimated yearly age‐related muscle loss in adults and estimated declines in total lean mass during the first year of IMD therapy in the STEP‐1 and SURMOUNT‐1 trials.^1^, ^2^, ^39^ Estimated declines in total lean mass during the 68‐week STEP‐1 and 72‐week SURMOUNT‐1 trials were normalized to 52 weeks based on the simplifying assumption that the decline in lean mass was linear over time. The estimated decline in muscle mass due to aging is based on numerous studies as described by Mitchell et al.^39^

In a pivotal trial of tirzepatide (Study of Tirzepatide in Participants with Obesity of Overweight; SURMOUNT‐1) for obesity, a subset of participants also had body composition analyzed by DXA before and at the end of the 72‐week treatment period (see supplementary appendix in Jastreboff et al.^2^). In the pooled tirzepatide‐treated (5, 10, or 15 mg once weekly) DXA subgroup, participants lost 10.9% of their lean mass during the treatment period. However, in the overall trial, tirzepatide‐treated patients lost 15% to 20.9% of their body weight. On a percentage basis, the loss of lean mass was similar to that reported among the semaglutide‐treated participants in the STEP‐1 trial. The estimated loss of skeletal muscle in these two major trials (10% or more during 68–72 weeks of treatment) approximates the average decline in muscle mass during 20 years of aging‐related muscle loss in adults older than 30 years, estimated at 3%–5% per decade (Figure 5).^39^

In earlier studies of modest weight reduction by non‐pharmacologic caloric restriction, declines in lean mass typically accounted for 10%–30% of the total body weight reduction.^6^ However, this percentage is not a constant and is expected to trend higher in people rapidly experiencing large weight reductions, as observed after bariatric surgery.^40^ It can also vary by sex, race, ethnicity, physical activity level, and other variables.^6^, ^41^ Although participants in the active‐treatment groups of the STEP‐1 and SURMOUNT‐1 trials experienced proportionately large declines in lean mass, those declines may be consistent with declines in lean mass expected in people experiencing large weight reductions.^40^ Or they could be due to characteristics of participants enrolled in the trials or other trial‐related factors. In the absence of additional studies or analyses, it is premature to speculate that the observed declines in lean mass were due to anything other than reduced caloric intake and possibly inadequate intake of specific macro‐ or micronutrients (in the context of low levels of physical activity).

Available evidence suggests that IMD therapy has beneficial effects on muscle structure and function in animal models and humans.^42^, ^43^, ^44^, ^45^, ^46^, ^47^, ^48^, ^49^, ^50^, ^51^ It is unclear whether those effects are sufficient to counteract the loss of muscle mass seen in clinical trials. However, there is evidence that exercise has beneficial effects when added to IMD therapy^52^ or to weight reduction induced by caloric restriction.^53^, ^54^, ^55^ It could be a decade or more before long‐term follow‐up studies evaluate the long‐term risk of sarcopenia or sarcopenic obesity associated with IMD therapy. Given the existing elevated risk in people with obesity,^7^ and taking into account current research gaps, efforts to minimize that risk should be a routine part of current obesity care.

## 3 PRESERVING MUSCLE MASS AND FUNCTION DURING IMD THERAPY FOR OBESITY

**FIGURE 6:**
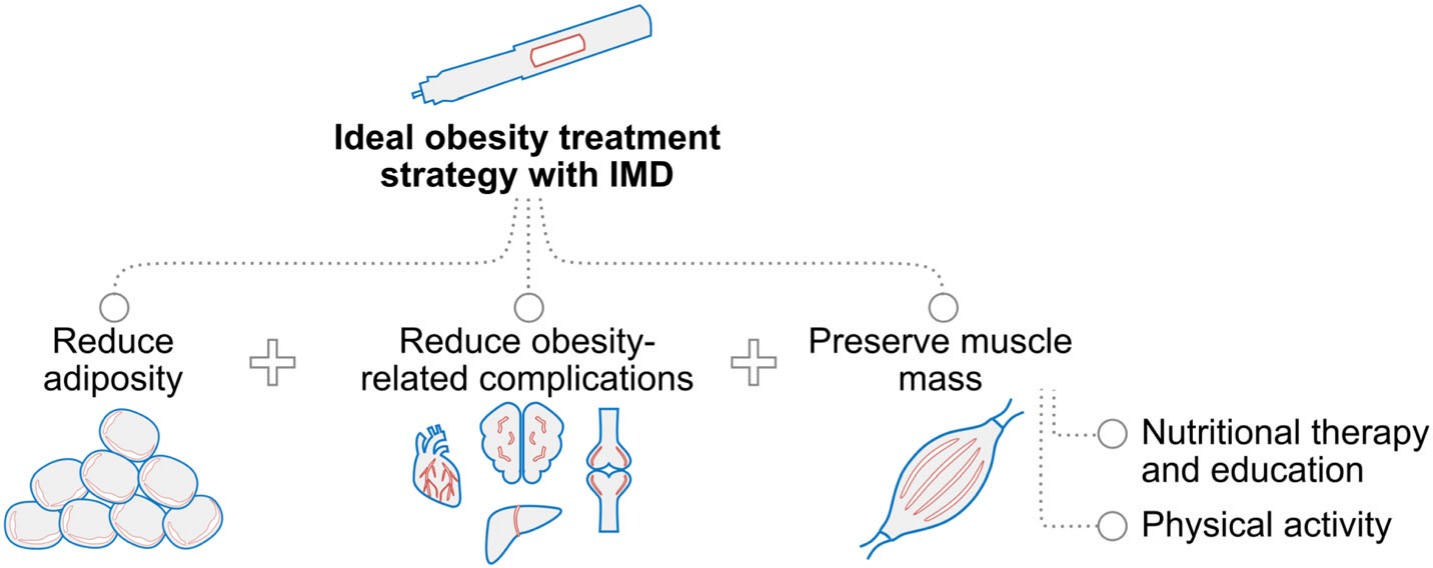
Comprehensive obesity management with incretin‐mimetic drugs. A comprehensive treatment strategy utilizing an IMD aims to reduce adiposity, mitigate obesity‐related complications, and preserve muscle mass. Treatment plans for preserving muscle mass should incorporate nutritional therapy and education alongside physical activity, with an emphasis on resistance training.

Although IMDs have pleiotropic effects,^5^ studies have demonstrated that reduced caloric intake accounts for much of their weight‐reducing effect when used for the treatment of obesity.^56^, ^57^, ^58^ It is often unrecognized that many people living with obesity have inadequate intake of protein and essential micronutrients.^59^, ^60^, ^61^ Therefore, the reduced caloric intake during IMD therapy may exacerbate pre‐existing nutritional deficits essential for the maintenance of muscle mass and function. Indeed, reduced muscle mass is now recognized as a phenotypic criterion for the diagnosis of malnutrition by the Global Leadership Initiative on Malnutrition^62^ and is also a defining characteristic of sarcopenia and frailty.^4^ If such nutritional deficits are accompanied by low levels of physical activity or a history of weight cycling, patients are at even greater risk of losing muscle mass and strength during IMD treatment. Thus, in the setting of obesity treatment with IMDs, preservation of muscle mass should be viewed as a key goal that parallels the goals of reducing adiposity and weight‐related complications (Figure 6). This principle is affirmed in the European consensus on the definition and diagnosis of sarcopenia.^28^, ^32^

### 3.1 Nutrition

Adequate nutrition is essential for the maintenance of muscle mass.^63^ Comprehensive obesity guidelines provide clear recommendations regarding macronutrient intake, a personalized approach to nutrition, and a comprehensive approach to management.^53^, ^54^, ^55^, ^64^ As noted in several guidelines, individualized nutrition education and medical nutrition therapy from a registered dietitian (RD) should be offered as core components of any obesity treatment plan.^53^, ^54^, ^55^, ^65^ If an RD is not accessible, the prescribing clinic should provide nutrition education and support.

In the setting of caloric restriction associated with IMD therapy, many patients may have inadequate protein intake. Thus, protein intake should be monitored to ensure it is adequate. Currently, the Institute of Medicine Recommended Dietary Allowance for protein in healthy people is 0.8 g/kg body weight/day.^66^ Higher amounts have been recommended for healthy people older than 65 years (1.2–1.5 g/kg body weight).^67^ Guidelines for the management of patients after bariatric surgery recommend a minimal protein intake of 60 g/day and up to 1.5 g/kg ideal body weight.^68^ Clinical nutrition guidelines from the Joslin Diabetes Center recommend protein intake of 1.0–1.5 g/kg of adjusted body weight, in which adjusted body weight is ideal body weight plus 0.25 × excess body weight.^69^ Protein intake should be adapted to meet the individual needs of each patient.^67^, ^68^, ^70^, ^71^ Furthermore, protein quality (as reflected by the content of essential amino acids and protein digestibility) affects the ability of dietary protein to support muscle protein synthesis.^72^ Thus, nutrition education and support should include individualized guidance on how patients can incorporate high‐quality protein sources in their diet.

Still unclear is the role of protein supplementation beyond recommended levels as a stand‐alone intervention to preserve muscle mass during caloric restriction or during maintenance of a reduced body weight. Studies addressing this issue have had mixed results, and many study designs make it difficult to apply the results to specific patient types such as people living with obesity on calorie‐restricted diets.^73^, ^74^ There are no current guidelines that recommend protein supplementation for these purposes beyond ensuring adequate protein intake on an individual basis.

Recent micronutrient guidelines emphasize that an adequate supply of all essential trace elements and vitamins (micronutrients) is essential for the metabolism of foods supplying protein and energy.^75^ Unfortunately, an analysis of National Health and Nutrition Examination Survey (NHANES) data suggests that more than 40% of US adults have inadequate intake of micronutrients.^61^ The recent micronutrient guidelines provide detailed information about the signs of micronutrient deficiency as well as how to assess and correct micronutrient levels.^75^ Another set of guidelines addressing nutrition after bariatric surgery also provides a reasonable template for a clinical approach to this issue.^68^

IMD therapy for obesity is associated with reduced appetite, temporary food aversion, and changes in taste preferences, especially during the initial stages of therapy.^76^ If patients receiving IMD therapy are unable to maintain a diet containing sufficient high‐quality protein and micronutrients, oral nutritional supplements (ONS) may be an effective and scalable option. With guidance from the clinical team, these products can be integrated into a comprehensive obesity treatment plan without undermining weight reduction efforts.^4^, ^54^ ONS provide protein, energy, and micronutrients for people who are not meeting their nutrition needs by food alone, and some are formulated with ingredients intended to support muscle health, as discussed in detail elsewhere.^4^ Use of ONS products is supported by extensive research and clinical guidelines.^77^, ^78^, ^79^ Note that dietary supplements are distinct from ONS and typically provide only specified types and amounts of individual nutrients, and they often have less research supporting their efficacy and safety.^80^, ^81^, ^82^, ^83^ Several randomized controlled trials have shown beneficial effects of ONS products on functional outcomes in various clinical scenarios,^84^, ^85^, ^86^ including significantly better leg strength in older (≥65 years), community‐dwelling adults at risk of malnutrition.^87^ Ongoing trials are evaluating the role of ONS to support muscle health in people with obesity.^88^

### 3.2 Physical activity

Both aerobic exercise and resistance training are widely recommended as essential components of a comprehensive obesity care plan.^53^, ^54^, ^55^, ^89^ Some guidelines recognize that resistance training can promote the maintenance of muscle mass and function during weight reduction therapy and promote weight maintenance after cessation of weight reduction therapy.^53^, ^54^, ^55^ In addition, several systematic reviews and meta‐analyses concluded that resistance training was effective for maintaining muscle mass during caloric restriction in people with overweight or obesity.^90^, ^91^, ^92^, ^93^ Another systematic review found similar results after bariatric surgery.^94^ These results are also supported by a randomized clinical trial of older adults living with frailty or reduced muscle mass induced by weight reduction.^95^ A study of maintenance therapy after low‐calorie weight reduction showed that exercise in combination with IMD therapy is associated with greater loss of adiposity and improvements in glycemic parameters and cardiorespiratory fitness versus IMD therapy alone or exercise alone.^96^ Furthermore, a long‐term follow‐up study of people with obesity who were treated with an IMD has shown that discontinuation of therapy was accompanied by weight regain as well as the return of cardiometabolic risk factors (e.g., high blood pressure and prediabetes).^11^ However, evidence suggests that when IMD therapy is accompanied by supervised exercise, those adverse effects occurring after treatment discontinuation can be attenuated.^52^

A program of physical activity that includes resistance training should take into account numerous patient‐specific factors including age, baseline fitness level, nutritional status, existing cardiovascular disease or risk factors, joint health and overall mobility, respiratory health, other comorbidities, medications, patient preferences, access to fitness equipment or resources, patient motivation, and psychological factors.^97^, ^98^ Guidance on the development of individualized goals and physical activity programs can be found in guidelines from the American College of Sports Medicine and the Physical Activity Guidelines for Americans.^97^, ^98^ The latter guidelines recommend at least 150–300 min per week of moderate‐intensity exercise or 75–150 min of vigorous‐intensity exercise as well as muscle‐strengthening activities on two or more days per week. It also notes the importance of working all the major muscle groups: legs, hips, back, abdomen, chest, shoulders, and arms.^97^ A practical consideration for developing resistance training plans is that muscle loss during weight reduction may affect some muscles more than others, and this effect may depend on the person's sex and age.^99^

A related question is whether a high‐protein diet increases the benefit of resistance training with respect to preserving muscle mass during caloric restriction in people with obesity. Several trials, systemic reviews, and meta‐analyses have addressed this question, but most of them studied older adults or people without obesity.^100^, ^101^, ^102^, ^103^ Because physical activity increases muscle protein synthesis, it is likely to increase protein needs. At the least, available studies reinforce the importance of maintaining adequate protein intake during caloric restriction in people with obesity.^32^ Other advantages of higher protein intake in this setting include increased satiety and energy expenditure, both of which contribute to weight reduction and maintenance of lower weight.^104^ Fortuitously, the two key strategies for preserving muscle mass and function during caloric restriction—nutrition and physical exercise—also have beneficial effects on bone,^105^, ^106^, ^107^ potentially reducing the risk for osteosarcopenic obesity.^108^

Skeletal muscle also functions as an endocrine organ. Muscle contraction during exercise triggers the release of numerous autocrine, paracrine, and endocrine mediators collectively known as myokines.^109^ These humoral factors have been shown to have beneficial effects on cognition, lipid and glucose metabolism, adipocyte function, bone structure, endothelial function, immune function, and skin structure.^109^ Though human research on this topic is limited, preclinical studies suggest that loss of muscle mass or function can detrimentally affect numerous physiological systems.^109^

## 4 ASSESSING MUSCLE MASS AND FUNCTION

**FIGURE 7:**
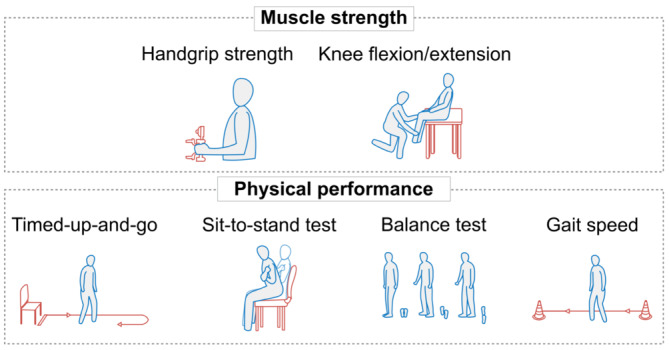
Methods for evaluating muscle strength and physical function.^28^ Adapted from Prado et al.^4^

Assessment of body composition (lean/muscle mass and fat mass) and muscle function can provide useful information to guide the clinical management of obesity.^110^, ^111^, ^112^ A summary of currently available methods is shown in Table 1. The most accurate and reliable methods for assessing body composition (magnetic resonance imaging [MRI], computed tomography [CT], and DXA) have limited availability in outpatient settings and relatively high costs.^62^ Although they should not replace the measurement of muscle mass, muscle function tests can be used as part of initial screening for suspected sarcopenic obesity (Figure 7).^28^ Declines in muscle function may be more evident than declines in muscle mass.^62^, ^116^ Currently, there is a critical need for studies exploring and validating how the results of body composition tests should guide clinical decision‐making during IMD therapy. For example, diagnostic cutoffs for many muscle function tests were developed and validated in older people and may not be applicable or accurate in younger populations.^16^

**TABLE 1:** Characteristics of selected methods for assessing body composition in outpatient settings.^a^

	Availability	Accuracy	Diagnostic performance	Longitudinal performance	Cost
MRI	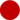	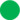	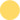	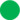	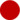
Ct	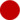	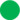	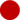	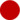	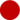
DXa	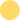	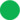	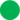	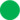	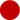
ADP	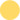	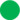	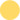	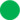	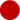
Ultrasound	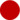	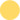	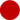	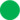	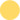
BiA	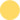	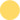	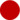	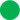	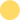
Anthropometry^b^	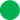	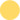	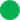	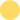	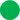

Abbreviations: ADP (BodPod), air displacement plethysmography; BIA, bioimpedance analysis; CT, computerized axial tomography; MRI, magnetic resonance imaging. Adapted from Prado et al.^4^

^a^ Diagnostic performance is limited by the availability of cutoff values. Longitudinal performance depends on test–retest reliability and follow‐up feasibility. Performance ratings are poor (red), moderate (yellow), or sufficient (green).

^b^ For example, mid‐upper arm circumference and calf circumference.^4^, ^110^, ^113^, ^114^ The latter is becoming more relevant for patients with obesity due to the availability of BMI‐adjusted measurements.^115^

## 5 PROSPECTS FOR THE FUTURE

Low muscle mass is a common and adverse feature of numerous diseases and conditions including obesity, sarcopenia, sarcopenic obesity, malnutrition, frailty, cardiovascular disease, T2D, and cachexia.^3^ Thus, there is considerable research interest in developing treatments to prevent muscle loss or wasting, including efforts focusing on adults with overweight or obesity. A recent study, for example, supports the concept of combining liraglutide therapy for obesity with exercise to improve the maintenance of weight reduction and metabolic parameters.^96^, ^117^ Effects on muscle mass remain to be explored, especially when exercise is combined with the greater weight reduction induced by more effective anti‐obesity IMDs. Other studies are exploring selective androgen receptor modulators^118^ or specific nutritional strategies intended to support muscle preservation including ingredients such as protein, amino acids, or β‐hydroxy‐β‐methylbutyrate (HMB), often as part of more complete ONS.^88^ Finally, recent studies and ongoing research support the promise of targeting alternative signaling pathways for treating obesity. For example, recent evidence suggests that agents targeting myostatin/activin signaling pathways may be able to effect reductions in adiposity while preserving or even increasing muscle mass.^119^, ^120^

## 6 CONCLUSIONS

During IMD therapy for obesity, preserving muscle mass and function is an essential treatment goal alongside reducing excess and abnormal adiposity. Two key principles for preserving muscle mass are (1) ensuring adequate intake of protein and other nutrients and (2) incorporating physical activity—specifically resistance training—into a comprehensive obesity treatment plan. ONS can counteract or prevent nutrient deficiencies and help patients maintain a balanced diet, especially in patients experiencing reduced appetite or food aversion. These nutritional strategies should be designed to provide patients with a targeted nutritional intervention without contributing to excessive caloric intake. These goals and principles reinforce the importance of patients having access to healthcare professionals who can offer evidence‐based guidance on nutrition and physical activity.

## AUTHOR CONTRIBUTIONS

This paper emerged from a scientific roundtable meeting in which all authors participated. The first draft was written by the medical writer on the basis of presentations and discussion at the scientific roundtable. All authors reviewed and revised the text and approved the final draft.

## CONFLICT OF INTEREST STATEMENT

JIM has received honoraria from Abbott Nutrition for lectures and serves on advisory boards for Abbott Nutrition, Aveta.Life, and Twin Health. WSB has received honoraria and/or paid consultancy from Novo Nordisk, Abbott Nutrition, Medscape, Alfie Health, and Med Learning Group. SMC has received honoraria and/or paid consultancy from Novo Nordisk, Eli Lilly, and Abbott Nutrition. OH has received research support from Eli Lilly and Novo Nordisk and serves on an advisory board for Abbott Nutrition. ZL attended the Abbott Nutrition Scientific Roundtable meeting. CMP has received honoraria and/or paid consultancy from Abbott Nutrition, Nutricia, Nestlé Health Science, Pfizer, and AMRA medical and investigator‐initiated grant funding from Almased. SBH has received honoria/paid consultancy from Medifast Corporation, Abbott Nutrition, Tanita Corporation, Novo Nordisk, Versanis, and Amgen.

## ROLE OF THE FUNDER/SPONSOR

Abbott Nutrition Health Institute funded the scientific roundtable meeting and manuscript preparation. Authors were reimbursed for the costs of attending the meeting but received no fee or honorarium for their role in the preparation of the manuscript. Abbott Nutrition employees did not influence the content or opinions expressed in the paper.
